# Genome-Scale CRISPR Screening Reveals Host Factors Required for Ribosome Formation and Viral Replication

**DOI:** 10.1128/mbio.00127-23

**Published:** 2023-02-21

**Authors:** Maikke B. Ohlson, Jennifer L. Eitson, Alexandra I. Wells, Ashwani Kumar, Seoyeon Jang, Chunyang Ni, Chao Xing, Michael Buszczak, John W. Schoggins

**Affiliations:** a Department of Microbiology, University of Texas Southwestern Medical Center, Dallas, Texas, USA; b Eugene McDermott Center for Human Growth and Development, University of Texas Southwestern Medical Center, Dallas, Texas, USA; c Department of Molecular Biology, University of Texas Southwestern Medical Center, Dallas, Texas, USA; d Department of Bioinformatics, University of Texas Southwestern Medical Center, Dallas, Texas, USA; e Department of Population and Data Sciences, University of Texas Southwestern Medical Center, Dallas, Texas, USA; Duke University School of Medicine

**Keywords:** RNA virus, flavivirus, ribosomes, translation, virus-host interactions

## Abstract

Viruses are known to co-opt host machinery for translation initiation, but less is known about which host factors are required for the formation of ribosomes used to synthesize viral proteins. Using a loss-of-function CRISPR screen, we show that synthesis of a flavivirus-encoded fluorescent reporter depends on multiple host factors, including several 60S ribosome biogenesis proteins. Viral phenotyping revealed that two of these factors, SBDS, a known ribosome biogenesis factor, and the relatively uncharacterized protein SPATA5, were broadly required for replication of flaviviruses, coronaviruses, alphaviruses, paramyxoviruses, an enterovirus, and a poxvirus. Mechanistic studies revealed that loss of SPATA5 caused defects in rRNA processing and ribosome assembly, suggesting that this human protein may be a functional ortholog of yeast *Drg1*. These studies implicate specific ribosome biogenesis proteins as viral host dependency factors that are required for synthesis of virally encoded protein and accordingly, optimal viral replication.

## INTRODUCTION

As obligate parasites, viruses depend on host cellular machinery for nearly every step of their replication cycles. Advances in genome-wide screening technologies enabled by HAP1 cells, small interfering RNA (siRNA) gene silencing, and CRISPR/Cas9 gene editing have elevated our knowledge of the host factor repertoire required by viruses. In many cases, these screens have identified host factors that are uniquely required for specific viruses. For example, CRISPR enabled identification of virus-specific entry factors CD300lf for norovirus (1) and the alphavirus receptor Mxra8 (2). Additionally, comparative screening performed with dengue, West Nile, yellow fever, hepatitis C, and Zika virus infections identified protein processing complexes required across the *Flaviviridae*, including the Sec61 translocon, the oligosaccharyltransferase (OST) complex, and endoplasmic reticulum (ER) membrane complex (EMC) (3–10).

In addition to virus family-specific host factors, host proteins that are more broadly utilized by diverse classes of viruses have also been identified through genetic screens. Examples include molecules involved in the vacuolar ATPase, heparan sulfate biosynthesis, endocytic trafficking, the conserved oligomeric Golgi (COG) complex, and translation initiation, among others (11–16).

Notably, host factor screens have not identified many proteins that regulate translation outside the initiation steps. These genes may have been missed because the screen relied on death-based selection strategies, which counterselect against essential host genes, or used siRNA strategies that fail to achieve complete gene silencing. Because co-opting translation machinery is a hallmark of all viruses, we hypothesized that additional host factors beyond those already identified may be important for viral protein expression and, by extension, genome replication.

Here, we used a genetically tractable flavivirus, yellow fever virus expressing the Venus green fluorescent protein, to implement a genome-scale CRISPR knockout screen that relied on virally encoded reporter gene expression as the selection strategy rather than cell survival after virus-induced cell killing. This approach revealed numerous known flavivirus host factors, as well as a cluster of 60S ribosome biogenesis factors that were not identified in previous screens. SBDS, a known ribosome biogenesis factor, and the relatively uncharacterized SPATA5 were validated in targeted knockout studies. We further demonstrate that these factors are broadly required for infection by multiple viruses representing 12 RNA and DNA viral families. Loss of each host factor affects polyribosome formation, thereby blunting replicative potential. SPATA5 was demonstrated to have a role in rRNA processing, implicating it as a functional ortholog to yeast *Drg1*. Together, these data identify previously uncharacterized viral host dependency factors and suggest a model in which specific proteins, some of which regulate ribosome biogenesis, are required to achieve a critical threshold of viral protein production required for optimal replication.

## RESULTS

**FIG 1:**
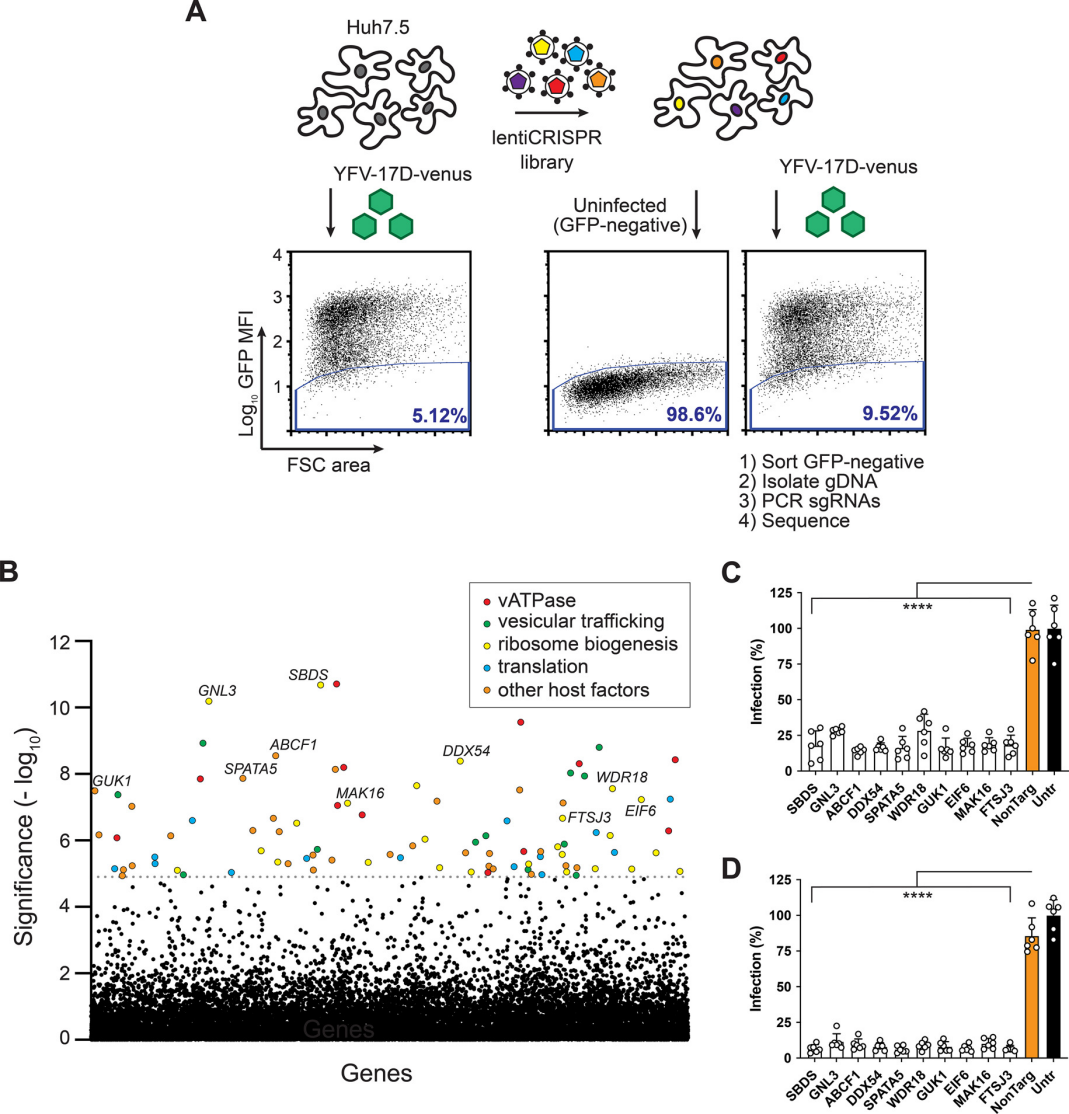
Yellow fever virus host factor CRISPR screen identifies 60S biogenesis host factors. (A) Schematic of genome-wide “negative-selection” CRISPR screen to identify host factors required for YFV replication. The blue line indicates the fluorescence-activated cell sorting (FACS) gate for green fluorescent protein (GFP)-negative cells. (B) Manhattan dot plot of CRISPR screen results with significance of enrichment calculated by MAGeCK. Genes with false-discovery rate (FDR) < 0.01 (dotted line) are colored. (C, D). Infectivity of Huh7.5 cells treated with sgRNAs targeting candidate genes infected with multiplicity of infection (MOI) 1 YFV-Venus for 24 h (C) or MOI 0.1 YFV-Venus for 48 h (D) and analyzed by flow cytometr. In panels C and D, *n* = 6 biological replicates. Statistical significance was determined by one-way analysis of variance (ANOVA). FSC, forward scatter; gDNA, genomic DNA; MFI, mean fluorescence intensity; NonTarg, nontargeting CRISPR guides; sgRNA, single guide RNA; Untr, untransduced control; ****, *P* < 0.0001.

The goal of this study was to implement a CRISPR screen to identify host factors required for virally encoded protein synthesis during a single replication cycle. To identify these putative host factors, we used a CRISPR-Cas9 “negative selection” strategy to enrich for single guide RNA (sgRNA)-expressing cells that failed to produce virally encoded Venus when infected with YFV-17D-Venus. In non-CRISPR-treated Huh7.5 cells, YFV-17D-Venus infected approximately 95% of cells, with 5% of cells remaining Venus-negative (Fig. 1A). However, in Huh7.5 cells transduced with the human CRISPR Brunello library (17), the percentage of Venus-negative cells during YFV-17D-Venus infection increased to ~9.5%, likely representing an enrichment of cells lacking genes required for any viral infection step up to and including virally encoded Venus expression. The Venus-negative cell population was collected by fluorescence-activated cell sorting (FACS), and significantly enriched sgRNAs were identified by MAGeCK. (18) (Fig. 1B; Table S1). Analysis of significantly enriched genes (false-discovery rate [FDR] < 1%) using the STRING database (19) revealed multiple pathways that have previously been identified in flavivirus host factor screens (3–11, 20–24). These include genes regulating the vATPase complex, ER-Golgi/vesicular trafficking, translation initiation, and heparan sulfate biosynthesis (Fig. S1A). We also identified 19 genes that mapped to a 60S ribosome biogenesis cluster that had not been identified in previous flavivirus studies, suggesting an important role for the 60S subunit in the YFV replication cycle.

10.1128/mbio.00127-23.1 FIG S1 60S biogenesis factors identified in CRISPR screen cluster by STRING. (A) STRING network plot of <1% false-discovery rate (FDR) CRISPR screen results generated by https://string-db.org. (B, C) YFV-Venus infectivity assayed by flow cytometry in A549 (B) or U-2 OS (C) control and host factor knockout (KO) cells (*n* = 4 biological replicates). Statistical significance was determined by one-way analysis of variance (ANOVA). *, *P* < 0.05; **, *P* < 0.01. Download FIG S1, PDF file, 2.8 MB. Copyright © 2023 Ohlson et al. 2023 Ohlson et al. https://creativecommons.org/licenses/by/4.0/ This content is distributed under the terms of the Creative Commons Attribution 4.0 International license.

10.1128/mbio.00127-23.7 TABLE S1 MAGeCK analysis of genome-wide CRISPR screen. Download Table S1, XLSX file, 1.9 MB. Copyright © 2023 Ohlson et al. 2023 Ohlson et al. https://creativecommons.org/licenses/by/4.0/ This content is distributed under the terms of the Creative Commons Attribution 4.0 International license.

To identify genes unique to our screen, we compared the <25% FDR candidate list to gene lists from flavivirus host factor screens performed using siRNA and CRISPR (3–5, 7–9, 11, 20, 21, 25). We selected for validation 10 high-scoring genes that had not been previously identified in other viral host-factor screens: SBDS, GNL3, ABCF1, DDX54, SPATA5, WDR18, GUK1, EIF6, MAK16, and FTSJ3. To confirm the role of each gene in supporting YFV replication, two distinct Brunello library sgRNAs targeting each gene candidate were cloned into either a puromycin- or a blasticidin-selectable lentiCRISPRv2 vector. Dual knockout (KO) Huh7.5 cells were generated by transducing cells with both lentiviruses and selecting cells in the presence of puromycin and blasticidin for 9 days to ensure high-efficiency gene knockout and protein depletion. When candidate KO and control cells were infected with a high multiplicity of infection (MOI) of YFV-17D-Venus for 24 h, or approximately one replication cycle, we observed a potent reduction in the percentage of infected KO cells compared to control cells (Fig. 1C). Similar results were obtained at low MOI and 48 h (Fig. 1D), confirming the requirement for these factors in the YFV replication cycle.

Seven of the ten factors (SBDS, GNL3, DDX54, WDR18, EIF6, MAK16, and FTSJ3) selected for follow-up were annotated in the STRING database to be 60S ribosome biogenesis factors, and therefore, the loss of each one may have contributed to impairment of viral infection via a common 60S subunit-based pathway. Since SBDS functions as the final cofactor in 60S maturation by removing EIF6 from the pre-60S particle (26), we chose it for further analysis as the representative 60S ribosomal biogenesis factor of the seven validated. We also chose the less well characterized protein SPATA5, as it contains two ATPase domains and resembles yeast Afg2/Drg1, which regulates yeast 60S biogenesis factor recycling (27). We first tested how loss of SBDS and SPATA5 affected YFV infectivity in two other human cell lines, A549 and U-2 OS. We observed reduced infection when these genes were targeted by CRISPR, indicating that the effects on YFV infection are not specific to Huh7.5 cells. (Fig. S1B and C).

**FIG 2:**
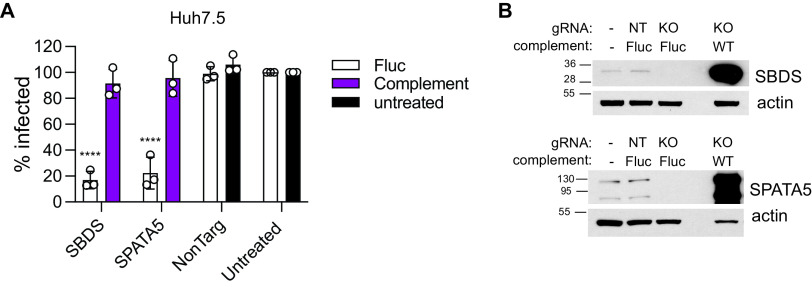
Genetic reconstitution of viral infection by expression of SBDS and SPATA5. (A) Infectivity of Huh7.5 cells treated with indicated CRISPR gRNAs and complemented with lentiviral expression of firefly luciferase (Fluc) or guide-resistant host factors infected with MOI 1 YFV-Venus for 24 h and analyzed by flow cytometry. (B) Lysates from Huh7.5 cells untreated or treated with indicated CRISPR gRNAs and complemented with lentiviral expression of Fluc or guide-resistant host factors were analyzed by Western blotting using anti-SBDS or anti-SPATA5 antibodies. Blot membranes were also probed with anti-actin antibodies (indicated) (*n* = 3 biological replicates). Statistical significance was determined by two-way ANOVA. KO, knockout; NonTarg or NT, nontargeting CRISPR guides; WT, wild type; ****, *P* < 0.0001.

To determine whether loss of SBDS or SPATA5 directly contributed to impaired viral infection, we reconstituted CRISPR-targeted cells with cDNAs expressing CRISPR-resistant versions of each host factor gene or firefly luciferase (Fluc) as a control. Herein, for simplicity, we refer to these CRISPR-targeted cells as knockout (KO) cells, with the caveat that these are bulk populations, not sequence-validated single cell clones. KO cells expressing Fluc control were unable to support high levels of YFV-17D-Venus infection, whereas KO cells that were complemented with each CRISPR-resistant host factor were as permissive to infection as control cells. (Fig. 2A). We used Western blot to confirm both CRISPR-mediated loss and cDNA-mediated reconstitution of each host factor (Fig. 2B). These results indicate that SBDS and SPATA5 are each specifically required for efficient YFV infection.

**FIG 3:**
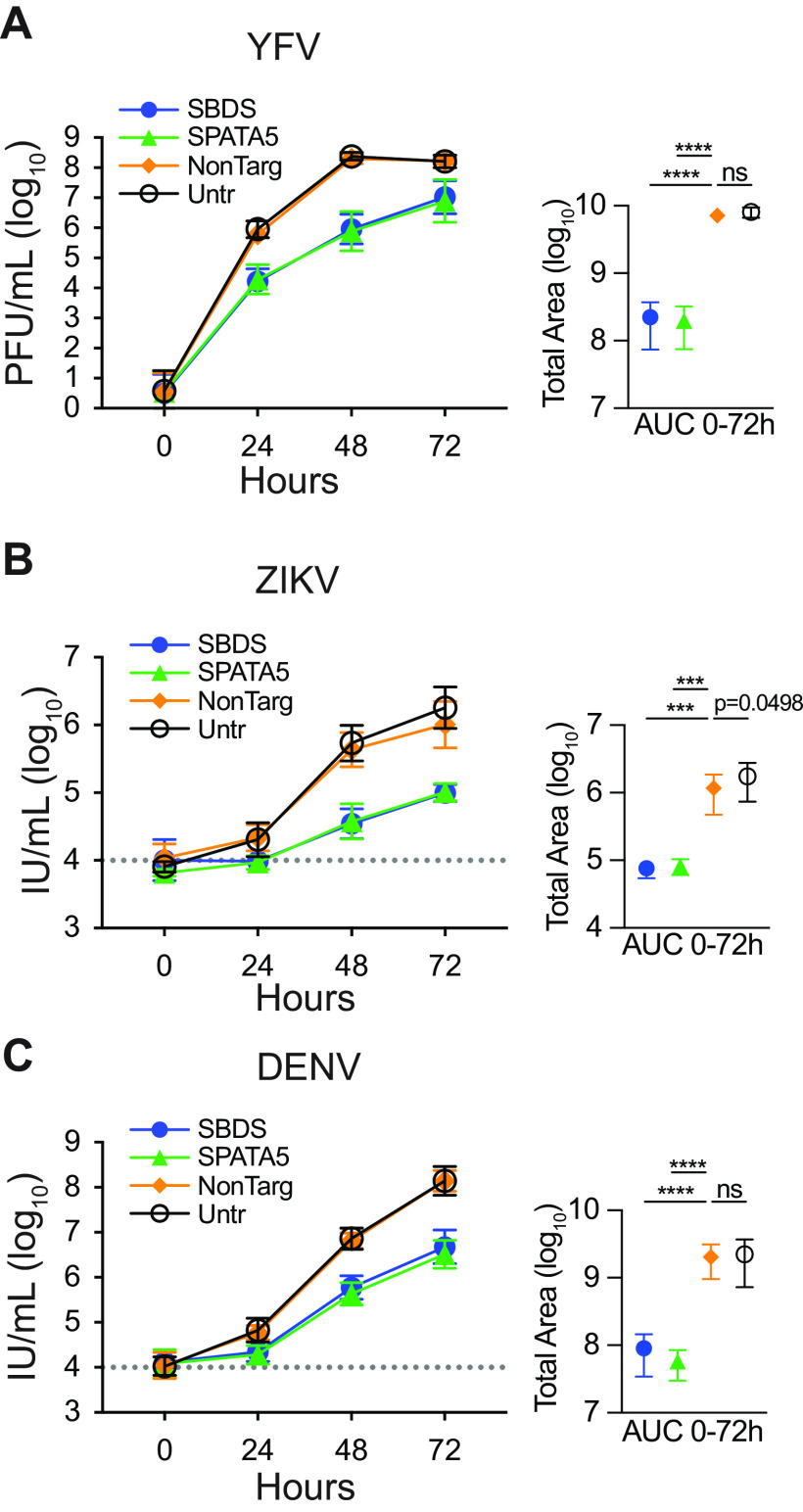
Flavivirus viral production is dependent on SBDS and SPATA5. (A to C) (Left panels) Viral growth curves from control or KO Huh7.5 cells infected with 0.01 MOI of YFV-17D quantified by plaque assay (A), 0.1 MOI Zika virus (ZIKV) PRVABC159 (B), or 0.1 MOI dengue virus (DENV2) (C). ZIKV and DENV2 titers determined by reinfection on susceptible Huh7.5 cells and antigen staining by flow cytometry. The area under the curve (AUC, right panels) was calculated and used to test statistical significance of each condition compared to NonTarg by one-way ANOVA (*n* = 4 biological replicates). ns, not significant; ***, *P* < 0.001; ****, *P* < 0.0001.

Since virally encoded Venus is a surrogate reporter for viral replication, we asked whether loss of SBDS or SPATA5 affected infectious viral particle production of the parental nonreporter virus YFV-17D. Control and KO Huh7.5 cells were infected with YFV-17D at 0.01 MOI, and virus production was quantified over time by plaque assay (Fig. 3A). Loss of either SBDS or SPATA5 resulted in log-fold reductions in YFV-17D titers. Similar results were obtained for the flaviviruses Zika virus (ZIKV) and dengue virus (DENV) (Fig. 3B and C), suggesting a broader requirement for these host factors in the *Flaviviridae* family.

**FIG 4:**
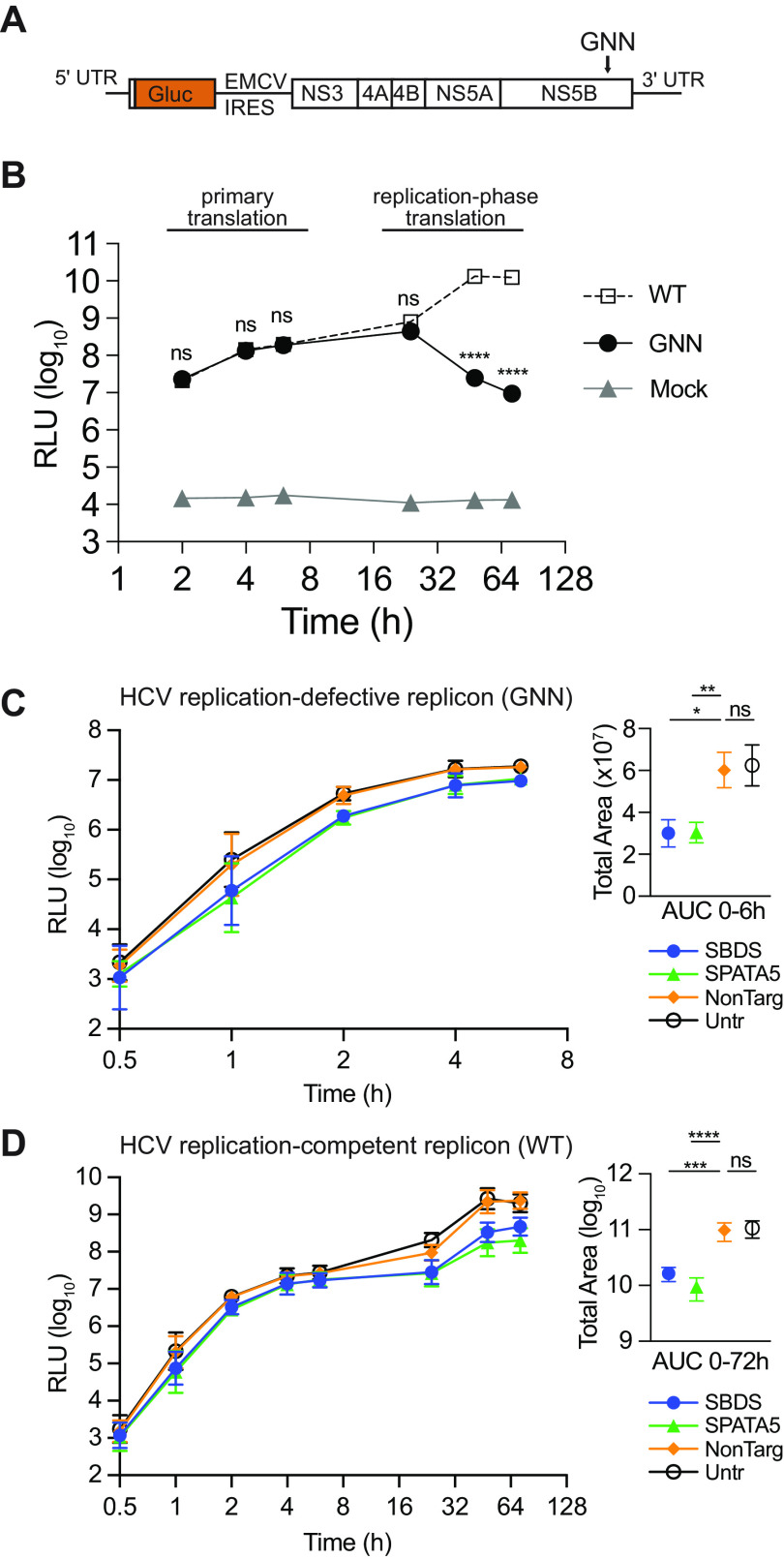
Virally encoded protein synthesis is reduced in SBDS and SPATA5 KO cells. (A) Cartoon of hepatitis C virus (HCV) subgenomic replicon. (B) Gaussia luciferase (Gluc) synthesis from a replication-defective (GNN) and replication-competent (WT) HCV subgenomic replicon in Huh7.5 cells. (C, D) (Left panels) Gluc synthesis from replication-defective GNN (C) or replication-competent WT (D) HCV subgenomic replicon in KO and control Huh7.5 cells. (Right panels) Area under the curve (AUC) was calculated and used to test statistical significance of each condition compared to NonTarg by one-way ANOVA (*n* = 4 biological replicates). NonTarg, nontargeting CRISPR guides; RLU, *Renilla* luciferase units; Untr, untransduced control; UTR, untranslated region; *, *P* < 0.05; **, *P* < 0.01; ***, *P* < 0.001; ****, *P* < 0.0001.

We next tested whether loss of SBDS or SPATA5 directly impaired synthesis of virally encoded proteins. For these assays, we used a replication-defective hepatitis C virus (HCV) replicon, which is a naked subgenomic RNA in which viral structural genes have been replaced with a secreted Gaussia luciferase (Gluc) reporter (Fig. 4A). The RNA-dependent RNA polymerase is also mutated (GDD→GNN), so that the Gluc signal is detected only from translation of the transfected RNA. In contrast, a wild-type replicon self-amplifies, leading to logarithmic increases in Gluc signal over time (Fig. 4B). When the GNN replicon was transfected into SBDS or SPATA5 knockout cells, we observed decreased Gluc synthesis at all time points (Fig. 4C). Area-under-the-curve analysis indicated that total Gluc levels were significantly reduced in the KO cells relative to nontargeting control. The differences between Gluc levels in control and KO cells were amplified considerably (2 to 3 log_10_) with the WT replicon at later stages of replication (Fig. 4D). This suggests that small defects in pioneering rounds of translation have pronounced consequences with respect to viral replication and virus production.

**FIG 5:**
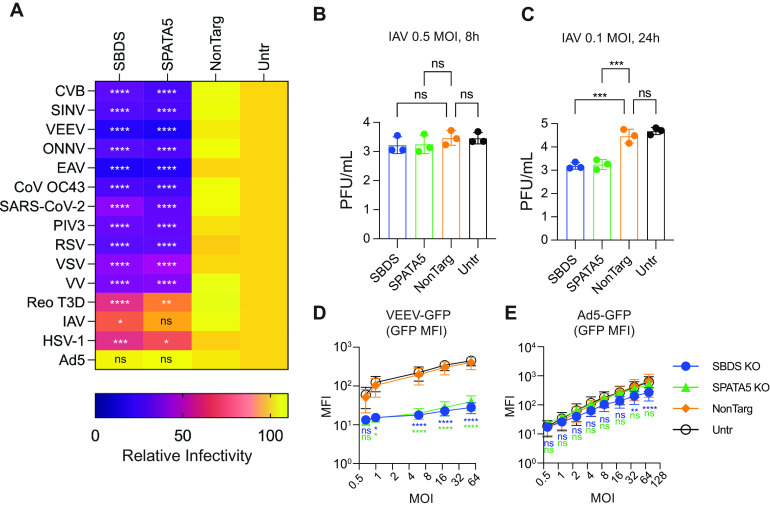
Diverse viruses are susceptible to host factor depletion. (A) Viral infectivity heat map of indicated viruses in control or KO Huh7.5 cells determined by flow cytometry. The data were normalized to untransduced control cells and represent three to five biological replicates. (B, C) Viral titers from supernatants from control or KO Huh7.5 cells infected with 0.5 MOI influenza A virus (IAV) for 7 h (B) or 0.1 MOI IAV for 18 h (C) determined by plaque assay. (D, E) GFP expression over a dose response of Venezuelan equine encephalitis virus (VEEV)-GFP (D) or Ad5-GFP (E) infection (*n* = 3 [B, C], *n* = 4 [D], or *n* = 6 [E] biological replicates). Statistical significance comparing nontargeting to SBDS KO or SPATA KO was determined by two-way ANOVA (A, D, E) or one-way ANOVA (B, C). NonTarg, nontargeting CRISPR guides; ns, not significant; Untr, untransduced control; *, *P* < 0.05; **, *P* < 0.01; ***, *P* < 0.001; ****, *P* < 0.0001.

We next tested whether the dependency of viruses on SBDS and SPATA5 extended beyond the *Flaviviridae*. We infected KO and control Huh7.5 cells with a panel of 15 diverse viruses, representing several negative- and positive-sense single-stranded RNA viruses, a double-stranded RNA virus, and double-stranded DNA viruses (Table S2). Viral infectivity was quantified by flow cytometry, using virally encoded green fluorescent protein (GFP) or virus-specific antigen staining. We observed that each host factor was required for optimal infection of cells by coxsackie virus B (CVB), equine arteritis virus (EAV), Sindbis virus (SINV), Venezuelan equine encephalitis virus (VEEV), O’nyong’nyong virus (ONNV), coronaviruses (CoV) OC43 and SARS-CoV-2, parainfluenza virus type 3 (PIV3), respiratory syncytial virus (RSV), vesicular stomatitis virus (VSV), and vaccinia virus (VV) (Fig. 5A; Fig. S2 and S3). Several viruses did not completely require SBDS or SPATA5, including influenza A virus (IAV), reovirus T3D (Reo), and herpes simplex virus-1 (HSV1). Ad5, a nonreplicating adenovirus vector expressing GFP from a cytomegalovirus (CMV) promoter, was largely unaffected by loss of host factors.

10.1128/mbio.00127-23.2 FIG S2 Normalized viral infectivity data used to generate the heat map in Fig. 5A. Viral infectivity of indicated viruses in control or KO Huh7.5 cells determined by flow cytometry measuring green fluorescent protein (GFP) or antigen staining. Download FIG S2, PDF file, 0.5 MB. Copyright © 2023 Ohlson et al. 2023 Ohlson et al. https://creativecommons.org/licenses/by/4.0/ This content is distributed under the terms of the Creative Commons Attribution 4.0 International license.

10.1128/mbio.00127-23.3 FIG S3 Non-normalized viral infectivity data related to Fig. S2. Viral infectivity (% GFP+) and viral gene expression (GFP or antigen mean fluorescence intensity [MFI]) were quantified by flow cytometry and plotted independently for each biological replicated. Download FIG S3, PDF file, 0.7 MB. Copyright © 2023 Ohlson et al. 2023 Ohlson et al. https://creativecommons.org/licenses/by/4.0/ This content is distributed under the terms of the Creative Commons Attribution 4.0 International license.

10.1128/mbio.00127-23.8 TABLE S2 Characteristics of viruses used in host factor knockout cells. Download Table S2, PDF file, 0.02 MB. Copyright © 2023 Ohlson et al. 2023 Ohlson et al. https://creativecommons.org/licenses/by/4.0/ This content is distributed under the terms of the Creative Commons Attribution 4.0 International license.

To examine why certain viruses were less affected by loss of SBDS or SPATA5, we used IAV as a model. In the assay presented above, we quantified IAV infectivity by NP staining during a single cycle of replication, and this was not significantly affected by loss of SBDS or SPATA5. We then tested whether dosing and time point factored into the observed phenotype with IAV. We quantified IAV PFU in control or KO cells at 0.5 MOI during one round of replication (Fig. 5B) or at 0.1 MOI over multiple rounds of replication (Fig. 5C). Only in the multicycle experiment did we observe reduced IAV titers in SBDS and SPATA5 KO cells relative to control. Similar to HCV replicon data, these results suggest that minor defects in virus replication may become amplified in KO cells after several rounds of replication. We also examined the impact of virus dose on the viral phenotype using VEEV-GFP, which was sensitive to loss of SBDS and SPATA5, and Ad5-GFP, which was relatively insensitive to loss of these host factors (Fig. 5A). Even at high doses, VEEV-GFP was still unable to overcome the loss of SBDS and SPATA5, as GFP levels were reduced greater than 10-fold at all doses relative to infected control cells (Fig. 5D). Conversely, GFP levels expressed from Ad5-GFP were similar in all cells at all but the highest dose of Ad5-GFP (MOI = 75) (Fig. 5E).

**FIG 6:**
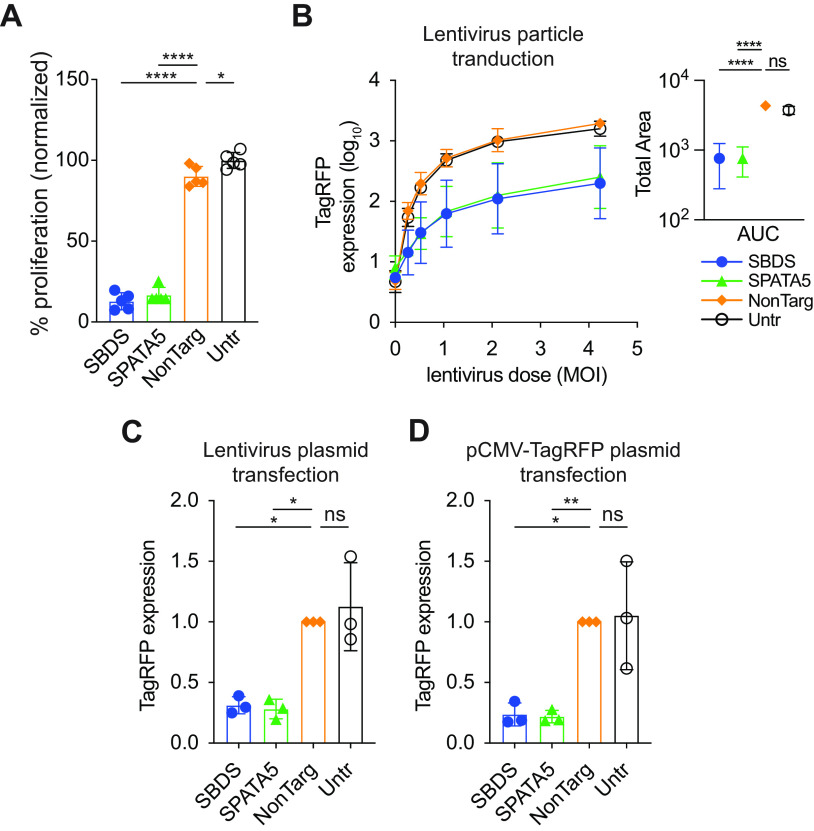
Host factor depletion affects cell health and heterologous reporter gene expression. (A) Control or KO Huh7.5 cells seeded overnight were assayed for proliferation by WST-1 assay. Statistical significance was determined by one-way ANOVA (*n* = 5). (B to D) flow cytometry-based quantitation of TagRFP expression in KO or control Huh7.5 cells transduced with TagRFP-expressing lentiviral particles (B), transfected with TagRFP-expressing lentiviral plasmid (C), or transfected with a plasmid driving TagRFP gene expression from a cytomegalovirus (CMV) promoter (D). In panel B, the area under the curve (AUC) was calculated from raw data (right panel) and statistical significance was tested by a one-way ANOVA. In panels C and D, data are presented as mean fluorescence intensity normalized to NonTarg control, and statistical significance was determined by ratio paired *t* test of the non-normalized data (*n* = 3). NonTarg, nontargeting CRISPR guides; Untr, untransduced control; *, *P* < 0.05; **, *P* < 0.01; ***, *P* < 0.001; ****, *P* < 0.0001.

As loss of SBDS and SPATA5 resulted in reduced replication for numerous viruses, we hypothesized that depleting these factors may affect cell health. We quantified proliferation rates and found that they were reduced ~5-fold in KO cells compared to control cells (Fig. 6A). Impaired cell viability and proliferation could be partially restored by complementing KO cells with CRISPR-resistant SBDS or SPATA5 cDNAs (Fig. S4A to C). We confirmed the complementation strategy by assessing restoration of VEEV-GFP in complemented cells (Fig. S4D and E). These results led us to test whether loss of SBDS and SPATA5 affected gene expression independently of viral replication or in a completely nonviral system. We transduced cells with a nonreplicating lentiviral vector expressing the red fluorescent protein TagRFP (SCRPSY-TagRFP). Alternatively, we transfected cells with the lentiviral backbone plasmid (pSCRPSY-TagRFP) or a naked DNA plasmid that expressed TagRFP from a CMV promoter. TagRFP reporter levels were quantified by flow cytometry. In all three cases, TagRFP expression was significantly reduced in SBDS and SPATA5 KO cells relative to control (Fig. 6B to D). These data indicate that the capacity for mRNA translation from viral or nonviral sources was compromised in KO cells, with viruses like Ad5-GFP and multicycle IAV deviating from this generalized effect.

10.1128/mbio.00127-23.4 FIG S4 Effects of SBDS KO and SPATA5 KO and gene complementation on cell health and Venezuelan equine encephalitis virus (VEEV)-GFP infection. (A) Timeline for generating KO cells, followed by gene complementation and assays to monitor cell health and viral infection. (B) Cell viability as measured by cellular ATP content (Cell Titer Glo assay). (C) Cell proliferation as measured by WST-1 assay. (D, E) Infection of VEEV-GFP in KO cell with and without gene-specific complementation. Infection was quantified as the percentage infected (% GFP+) or viral reporter expression (mean GFP MFI) by flow cytometry (*n* = 3 biological replicates). Statistical significance between firefly luciferase (Fluc)-complemented or SBDS/SPATA5-complemented cells was determined by unpaired *t* test. IRES, internal ribosome entry sites; ns, not significant; *, *P* < 0.05; **, *P* < 0.01; ***, *P* < 0.001. Download FIG S4, PDF file, 0.5 MB. Copyright © 2023 Ohlson et al. 2023 Ohlson et al. https://creativecommons.org/licenses/by/4.0/ This content is distributed under the terms of the Creative Commons Attribution 4.0 International license.

**FIG 7:**
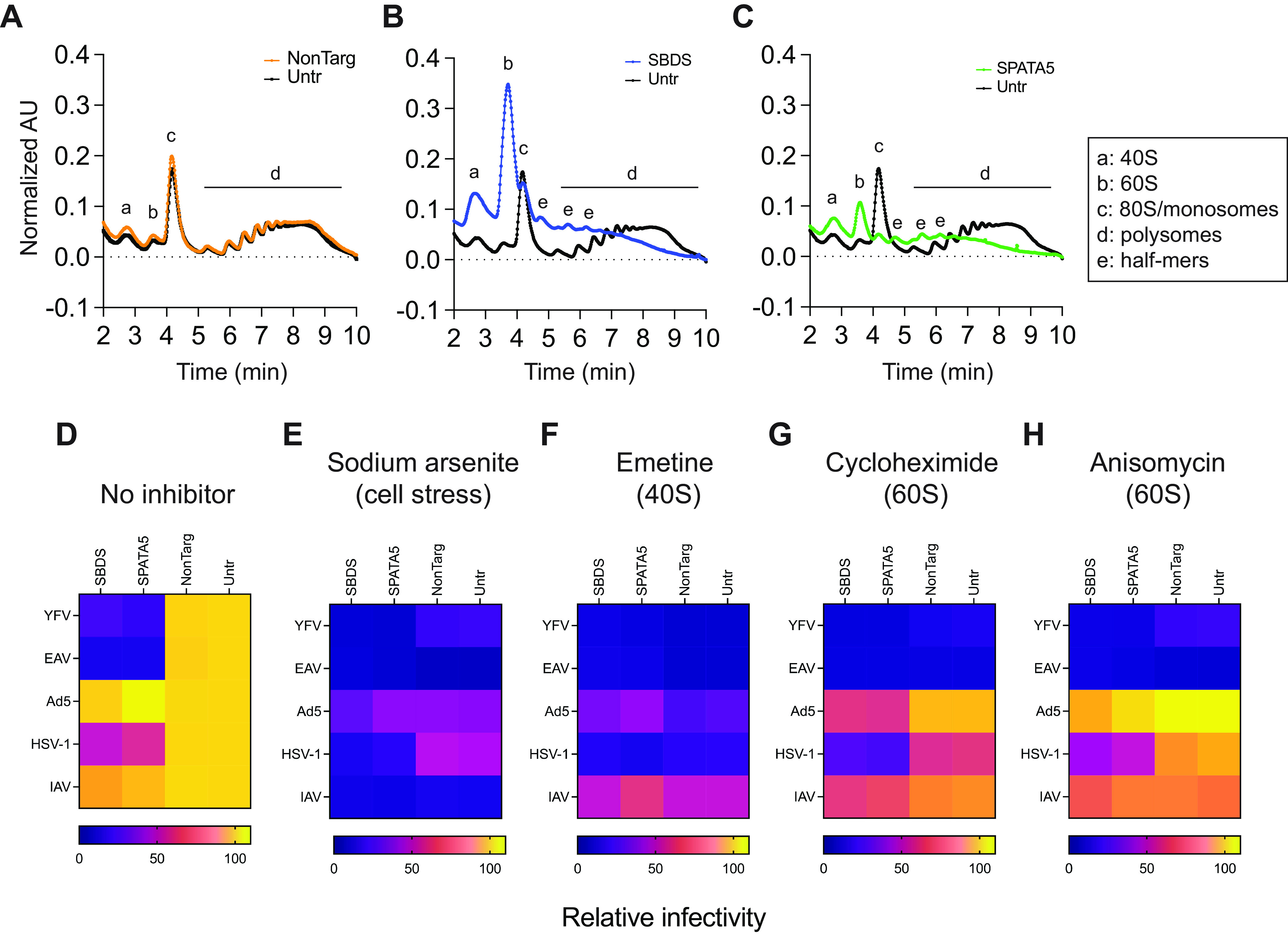
Host factor knockout cells lack high-molecular weight polysomes. (A to C) Sucrose density gradient ribosome fractionation profiles of nontargeting control (A), host factor KO cells SBDS (B), or SPATA5 (C) overlaid with WT untreated Huh7.5 cell profiles (black). (D to H) Viral infectivity heat maps of control and KO Huh7.5 cells infected with cytoplasmic (YFV, equine arteritis virus ([EAV]) or nuclear viruses (Ad5, HSV-1, IAV) for 1 h prior to treatment without (D) or with the following inhibitors: 50 μM sodium arsenite (E), 2 μM emetine (F), 2 μM cycloheximide (G), or 10 μM anisomycin (H). Infectivity determined by flow cytometry. NonTarg or NT, nontargeting CRISPR guides; Untr, untransduced control; AU, absorbance units.

These global effects on cell health, combined with the requirement of these host factors for both viral and nonviral protein synthesis, led us to examine the effect of host factor depletion on cellular ribosome profiles. Lysates of uninfected control and SBDS or SPATA5 KO cells were subjected to sucrose-gradient centrifugation and fractionation. Nontargeting control cells had a polysome profile identical to WT cells (Fig. 7A). In contrast, SBDS KO cells had increased 40S and 60S peaks, higher levels of low-molecular-weight (LMW) polysomes, and lower levels of high-molecular-weight (HMW) polysomes (Fig. 7B). Similar polysome profiles have been observed in cells bearing autosomal recessive SBDS mutants (28). Additionally, SBDS KO LMW polysome peaks included “half-mer” peaks that likely contain a 47S preinitiation complex lacking a joined 60S (29) and a variable number of ribosomes, suggesting a defect in 60S-joining activity. The SBDS KO profile is consistent with a lack of 60S maturation as the increased 60S peak likely contains pre-60S subunits that are unable to form a competent 80S. Similar to SBDS KO, the SPATA5 KO profile had increased 40S and 60S peaks, reduced 80S, increased LMW polysomes including half-mers, and lower HMW polysomes (Fig. 7C).

The polysome profiles indicate that depletion of SBDS and SPATA5 may result in specific defects in the 60S particle or its ability to form an 80S monosome. Accordingly, we hypothesized that chemical inhibitors targeting specific stages of translation would phenocopy the viral susceptibility phenotypes of KO cells. Four translation inhibitors with different mechanisms of action were chosen: sodium arsenite induces cell stress (30), emetine binds the 40S subunit (31), cycloheximide binds the 60S E site, and anisomycin binds the 60S A site (32). Conditions for each inhibitor were selected that did not cause pronounced host toxicity over a 24-h treatment period. We do note, however, that the dose of sodium arsenite used did result in a modest reduction in cell viability (Fig. S5). We then quantified viral replication/gene expression with and without inhibitor treatments 1 h postinfection for viruses with differing dependencies on SBDS and SPATA5 (YFV and EAV have strong dependency; HSV-1 and IAV have moderate dependency; and nonreplicating Ad5 vector has weak dependency.) Without inhibitor treatment, KO cells were more refractory to YFV and EAV than to Ad5, HSV-1, and IAV, as observed above (Fig. 7D). Cell stress-induced translation inhibition by sodium arsenite reduced replication of all viruses in control and KO cells (Fig. 7E). Similarly, blocking translation initiation on the 40S subunit by emetine also inhibited all viruses (Fig. 7F). However, inhibition of translation elongation by targeting the 60S subunit with either cycloheximide or anisomycin treatment reduced replication of YFV and EAV, both strongly dependent on SBDS and SPATA5, to a much greater extent than the other viruses (Fig. 7G and H). The patterns of inhibitor susceptibility correlate with KO cell viral replication phenotypes and support the hypothesis that, under the experimental conditions used here, viruses with strong sensitivities to SBDS and SPATA5 depletion may have a unique dependence on the 60S subunit compared to viruses that are less affected.

10.1128/mbio.00127-23.5 FIG S5 Effects of translation inhibitors on cell viability. ATP levels of untreated or treated cells levels were determined by Cell Titer Glo assay (*n* = 3 biological replicates). Statistical significance between untreated controls and drug-treated conditions was determined on log-transformed data by two-way ANOVA. ns, not significant; *, *P* < 0.05; **, *P* < 0.01. Download FIG S5, PDF file, 0.4 MB. Copyright © 2023 Ohlson et al. 2023 Ohlson et al. https://creativecommons.org/licenses/by/4.0/ This content is distributed under the terms of the Creative Commons Attribution 4.0 International license.

As a correlate of this hypothesis, we examined the polysome profile of nonreporter and Venus-expressing YFV-infected Huh7.5 cells. With infection, polysomes were nearly absent, and we observed a concomitant increase in 80S monosomes compared to uninfected cells (Fig. S6A). An additional increase in the 60S subunit peak, but not the 40S subunit peak, was observed in infected cells. Polysome collapse has previously been observed with several other viruses that also depend on SBDS and SPATA5, including the flaviviruses (DENV) and West Nile virus (WNV) (33), the rhabdovirus VSV (34), and poliovirus, a picornavirus related to SBDS/SPATA5-sensitive CVB3 (35). Intriguingly, infection of cells with IAV and Ad5, both of which were largely unaffected by loss of SBDS and SPATA5, does not cause polysome collapse or other significant changes to polysome profiles (36, 37).

**FIG 8:**
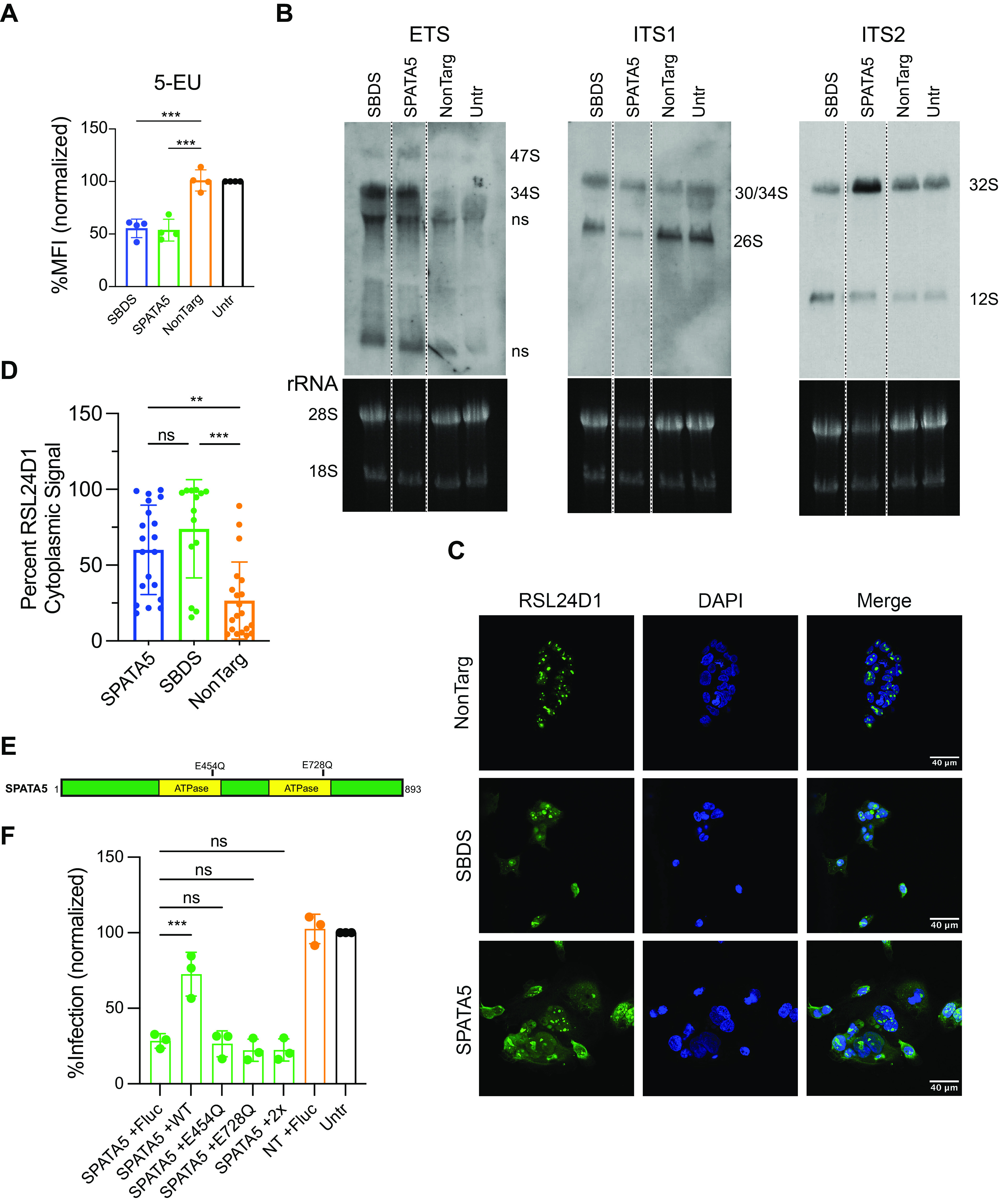
SBDS and SPATA5 regulate ribosome biogenesis. (A) Normalized MFI of control or KO Huh7.5 cells treated with 5-ethynyl-uridine (5-EU), labeled by click chemistry, and analyzed by flow cytometry. (B) Northern blots of control or KO cell RNA labeled with ETS, ITS1, and ITS2 probes. Images of rRNA gels prior to membrane transfer are shown below blots. Band sizes of rRNA species are labeled. (C) Control nontargeting, SBDS KO, or SPATA5 KO Huh7.5 cells were stained with an anti-RSL24D1 antibody (green) and 4′,6-diamidino-2-phenylindole (DAPI) (blue) and imaged by confocal microscopy. (D) Quantification of cytoplasmic localized RSL24D1 in control nontargeting, SBDS KO, or SPATA5 KO Huh7.5 cells. Quantifications were done with three separate transductions of cells except SBDS, which was done with two preparations. At least six image fields were quantified per cell line per preparation. (E) SPATA5 protein domain cartoon indicating catalytic residues targeted by site-directed mutagenesis. (F) YFV-Venus infectivity assayed by flow cytometry in control or KO cells transduced with lentivirus expressing wild-type or mutant SPATA5 (F) or Fluc control. NonTarg or NT, nontargeting CRISPR guides; ns, nonspecific; Untr, untransduced control.

10.1128/mbio.00127-23.6 FIG S6 Data supporting polysome profiling and mechanistic studies presented in Fig. 7 and 8. (A) Sucrose-density gradient ribosome fractionation profile of uninfected Huh7.5 cells (black) or infected with MOI 5 YFV-17D (red) or YFV-Venus (green) viruses for 24 h. (B) Schematic of Northern blot probe binding sites in rRNA. (C) Expression of guide-resistant SPATA5 in SPATA5 KO cells. Lysates of Huh7.5 SPATA5 KO cells complemented with lentivirus-expressed WT or catalytic mutant guide-resistant SPATA5 were analyzed by Western blotting using anti-SPATA5 and anti-actin antibodies. Download FIG S6, PDF file, 0.8 MB. Copyright © 2023 Ohlson et al. 2023 Ohlson et al. https://creativecommons.org/licenses/by/4.0/ This content is distributed under the terms of the Creative Commons Attribution 4.0 International license.

Together, our data suggest that in KO cells, viral protein production can achieve only a certain threshold, or “ceiling.” If that threshold is not reached, as it is in wild-type cells, viral replication is suboptimal. This limited protein production correlates with defects in the ribosome profiles of KO cells. One possibility is that the affected viruses require *de novo* ribosome formation. For example, RPL40-containing ribosomes have been shown to be required for optimal VSV infectivity (38). To determine whether *de novo* ribosome production may be affected, we treated control and KO cells with the alkyne-containing 5-ethynyl-uridine (5-EU). 5-EU is a uridine mimic that is incorporated into all RNA, but the high abundance of rRNA relative to other RNAs allows 5-EU to be used a surrogate for potential effects on rRNA production. SBDS and SPATA5 KO cells were 5-EU-positive, but they exhibited lower 5-EU incorporation compared to control cells (Fig. 8A), suggesting that *de novo* production of ribosomal rRNA may be reduced. To determine whether KO cells had defects in the maturation of *de novo* ribosomes, we assessed rRNA processing by Northern blotting, with probes that detect specific forms of maturing rRNA as ribosomes are formed (39). Loss of SBDS and SPATA5 resulted in increased 47S and 34S RNAs, suggesting defects of early cleavage reactions in the 5′-ETS (Fig. 8B; Fig. S6B). Cells lacking SPATA5 additionally accumulated 32S pre-RNA, suggesting impaired ITS2 processing. In yeast, when 60S ribosome biogenesis is disrupted, certain ribosome biogenesis proteins such as Rpl24 are retained with pre-60S particles and thus have defective nuclear shuttling. To determine whether KO cells display altered biogenesis factor localization, we assessed localization of RSL24D1, the human homolog of yeast Rpl24, by immunofluorescence and confocal microscopy. SBDS and SPATA5 KO cells accumulated more RSL24D1 in the cytosol than control cells, suggesting aberrant recycling of RSL24D1 in the absence of these proteins (Fig. 8C and D). These data complement polysome profiling and inhibitor studies to suggest that SBDS and SPATA5 are involved in the maturation of ribosomes required for optimal viral protein synthesis. The phenotypes of the SPATA5 knockout cells are also consistent with the reported role of the SPATA5-like yeast protein Drg1 in regulating 60S maturation (40). Drg1 has two ATPase domains, and its ATPase activity is required for regulating 60S formation. To test whether the enzymatic activities SPATA5 are required for viral infection, we inactivated ATPase activity by introducing single (glutamic acid to glutamine) and double (2×) Walker B mutations in the two ATPase domains (Fig. 8E). Wild-type or enzymatic mutants of SPATA5 were expressed in SPATA5 KO cells (Fig. S6C). In contrast to WT SPATA5, the mutant proteins failed to complement YFV infection (Fig. 8F), indicating that that viral infectivity depends on catalytically active SPATA5. Together, our data provide evidence to suggest that human SPATA5 is a functional ortholog of yeast Drg1.

## DISCUSSION

In this study, we used a FACS-based negative selection CRISPR screening approach to identify SBDS and SPATA5 as important regulators of a diverse panel of viruses. We discovered that loss of these host factors impairs ribosome formation and viral protein synthesis, leading to reduced viral replication. Loss of these host factors also had negative effects on cell proliferation. Viral sensitivity to host factor loss paralleled phenotypes produced by treatment with translation inhibitors that target the 60S subunit of the ribosome complex, suggesting that that some viruses may have specific ribosome requirements for replication. Alternatively, viruses that are unaffected by loss of SBDS and SPATA5 may have their own mechanisms to overcome the translation defect, as discussed further below.

In the CRISPR screen, 19 of the top 93 (<1% FDR cutoff) host factor candidates were annotated to 60S ribosome biogenesis factors, and 7 were large subunit (RPL) proteins. In contrast, no 40S biogenesis factors or small subunit (RPS) proteins were found in the top <1% FDR. Expanding the candidate list to the top <5% FDR, a total of 16 RPL and 3 RPS candidates are present, emphasizing the critical role that 60S and RPL proteins may play in flavivirus replication. Indeed, the importance of individual RPL proteins for viral infectivity has been demonstrated for viruses across divergent host species; mammalian RPL40 is important for VSV infection (38), Aedes aegypti RpL23 and RpL27 are required for ZIKV replication in mosquito cells (41), and several Saccharomyces cerevisiae 60S RPLs are required to maintain L-A virus in yeast (29).

Viruses have evolved numerous strategies to hijack their host’s translational machinery. For example, some viruses use specific RNA structures to form internal ribosome entry sites (IRES) to initiate translation, while other viruses use methods to steal host mRNA components to mimic cellular mRNAs. The viruses that we found to be sensitive to loss of SBDS and SPATA5 do not have any one single translation-hijacking strategy in common. Additionally, not all viruses were affected equally, with nonreplicating Ad5 vector being the least sensitive to loss of SBDS and SPATA5. IAV in a single-cycle assay was also unaffected by loss of SBDS and SPATA5. These findings raise the possibility that the viruses sensitive to depletion of SBDS or SPATA5 may be directly affected by specific changes to ribosomes (likely 60S) and/or indirectly affected by downstream effects on cell health. Indeed, loss of SBDS has been reported to alter cell sensitivity to various stressors by mechanisms that are thought to be independent of its role as a ribosome biogenesis factor (42). SBDS is also a very stable protein (43). Thus, to uncouple a transient ribosome-specific defect from longer-term effects on cell proliferation, our future studies might benefit from an experimental system in which could rapidly induce SBDS and SPATA5 turnover at the protein level, perhaps via an auxin-inducible degron system.

A mechanism to explain why viruses like Ad5-GFP are relatively insensitive to loss of SBDS and SPATA5 is that these viruses may have strategies to compensate for the deficiencies in the KO cells. For example, SBDS plays a critical role in ribosome maturation by promoting the release of EIF6, a host factor that prevents premature association of the 60S particle with the 40S subunit (44). In an SBDS-deficient state, EIF6 is not efficiently released, and the 60S particle is not available for ribosome formation (45). In one study, a structural inner capsid protein, protein VIII from bovine adenovirus 3, was found to interact with EIF6 (46). The authors of that study speculated that the protein VIII-EIF6 interaction may facilitate preferential translation of adenovirus genes. Future studies are needed to corroborate this model with human adenovirus and to examine whether other viruses that tolerate loss of SBDS and SPATA5 have similar strategies to manage ribosome maturation defects.

Our viral phenotypes, polysome profiling, drug studies, and mechanistic data suggest a link between loss of mature 60S subunits and diminished viral translation. Loss of SBDS in host cells likely produces a pool of pre-60S subunits that are unable to form mature 80S monomers due to lack of EIF6 removal from pre-60S subunits that have been exported from the nucleus (47). The AAA ATPase SPATA5 shares homology with yeast Afg2/Drg1, which has been shown to regulate the release and recycling of 60S maturation factors. In both SBDS and SPATA5 KO cells, the presence of half-mers and an increased 60S peak by ribosomal fractionation suggest an accumulation of pre-60S subunits (Fig. 7B and C), which may outnumber mature, 80S competent subunits. Notably, in a recent study, SPATA5 was independently discovered along with C1ORF09 to have a similar role in maturation of pre-60S subunits (48). C1ORF09 was a statistically significant hit in our CRISPR screen but was not selected for follow-up studies.

In summary, these studies demonstrate that by technically revamping more traditional virus-focused CRISPR screens, previously uncharacterized viral host dependency factors can be identified, revealing new insight into the requirement of specific host proteins for protein synthesis and viral replication. Our data indicate that viruses may have specific requirements for 60S ribosome biogenesis factors to achieve optimal viral protein synthesis and replication. It is tempting to speculate whether transient depletion of these host factors could lower the ceiling of maximal viral protein synthesis, thereby imparting therapeutic antiviral effects. Indeed, a recent study demonstrated this proof of concept by inhibiting SARS-CoV-2 translation with plitidepsin, which targets the translation factor eEF1A (49).

## MATERIALS AND METHODS

### Cell lines.

A549, U-2 OS, HEK293T, MDCK, and Huh7.5 cells (from C. Rice, Rockefeller University) and all derivatives were grown in Dulbecco’s modified Eagle’s medium (DMEM) (Gibco) supplemented with 10% fetal bovine serum (FBS) and 1× nonessential amino acids (Gibco). BHK-21J cells were grown in minimum essential medium (MEM) supplemented with 10% FBS and 1× nonessential amino acids. L929 cells were grown in DMEM supplemented with 5% FBS, 1% penicillin-streptomycin (Sigma), and 0.1% amphotericin B (Fisher Scientific). All cells were maintained at 37C in 5% CO_2_. Cells expressing selectable markers were grown in complete media supplemented with puromycin (Sigma) ranging from 0.5 to 4 μg/mL or blasticidin (Gibco) ranging from 10 to 20 μg/mL, depending on the cell line. The cell lines were tested for mycoplasma using a PCR assay (Venor GeM mycoplasma detection kit, Sigma).

### Viruses.

The generation and production of the following viruses have been previously described (50–54): YFV-17D, YFV-17D-Venus, CVB-GFP, SINV-GFP, DENV-GFP, ZIKV-GFP, HCV-Gluc, PIV3-GFP, RSV-GFP, VSV-GFP, EAV-GFP, VEEV-GFP, VV-GFP, ONNV-GFP, and WSN IAV. Reovirus type 3 Dearing (T3D) was provided by T.S. Dermody, and stocks were generated in L929 cells, gradient purified, and quantified by plaque assay in L929 cells as previously described (55–57). Ad5-GFP was propagated in HEK-293 cells. HSV1-GFP strain 17 (provided by D. Leib) and SARS-CoV-2 (USA-WA1/2020 BEI No. NR-52285) were propagated in VeroE6 cells. Coronavirus OC43 (ATCC VR-1558) was propagated in HCT-8 cells and detected by antibody staining (MAB9012, Millipore).

### Lentivirus pseudoparticle production and transduction.

LentiCRISPRv2 (puromycin, a gift from Feng Zhang, No. 52961) (58) and lentiCRISPRv2-BLAST (a gift from Mohan Babu, No. 83480) were purchased from Addgene. Oligonucleotides encoding single guide RNA sequences (Table S3) were annealed and ligated into LentiCRISPRv2 vectors linearized with Esp3I according to Addgene protocol. The SCRPSY-TagRFP and SCRBBL lentiviral vector for overexpression have been previously described (53, 59). Infectious lentivirus was produced by cotransfecting plasmids encoding VSV-g, Gag-Pol, and lentivirus vectors at a 2:8:10 μg ratio to HEK293T cells using Xtreme-Gene9 (Roche), and lentivirus-containing supernatants were collected 72 h post-transfection. For generating pools of single-gene knockouts, the cells were reverse-transduced by plating cells in the presence of lentivirus expressing two different gene-targeting sgRNAs for 48 h, and then the cells were grown in the presence of selective media for 3 to 12 days prior to seeding for experiments.

10.1128/mbio.00127-23.9 TABLE S3 Oligonucleotides and probes used in this study. Download Table S3, XLSX file, 0.01 MB. Copyright © 2023 Ohlson et al. 2023 Ohlson et al. https://creativecommons.org/licenses/by/4.0/ This content is distributed under the terms of the Creative Commons Attribution 4.0 International license.

### CRISPR-Cas9 host factor screening.

Human Brunello CRISPR knockout pooled library was a gift from David Root and John Doench (Addgene No. 73178) and amplified according to instructions. Production of library lentivirus and transductions of Huh7.5 cells were performed as previously described (53). Huh7.5 cells transduced with 100× coverage of CRISPR library lentivirus were amplified and passaged for 2 weeks prior to screening: 1 week in complete DMEM supplemented with 4 μg/mL puromycin followed by another week in complete DMEM without antibiotics. The day before infection ~8 × 10^6^ library cells/15-cm plate were seeded to six plates. Each plate was infected with YFV-Venus (MOI 5) in 16 mL of DMEM supplemented with 1% FBS for 3 h, then 10 mL of complete DMEM was added, and the plates were incubated overnight. Twenty-four hours postinfection, infected library cells were rinsed with phosphate-buffered saline (PBS), harvested with trypsin, and pooled. The pelleted cells were resuspended in PBS supplemented with 2% FBS and 0.5 mM EDTA, filtered by a 100-μm filter, and stored on ice before sorting at the Children’s Medical Center Research Institute Flow Cytometry Facility using a FACSAria II (Becton Dickenson) flow cytometer. GFP-negative gated cells were collected in PBS supplemented with 50% FBS and 50 mM HEPES and pelleted. Genomic DNA (gDNA) was extracted from sorted cell pellets as previously described (53). Uninfected library cells (3 × 10^6^) were harvested, and gDNA was extracted for controls. The entire screen procedure was performed three separate times, and each time, 3.5 to 5.4 × 10^7^ cells were sorted by FACS, representing an average of 600-fold library coverage for each screen. Library amplicons of sgRNA sequences in each gDNA sample were generated by PCR using 6 to 10 μg gDNA per 100-μL PCRs (60). Each sample was amplified in four parallel 100-μL PCRs using barcoded P7 primers (Table S3), then pooled, and purified by AMPure XP beads (Agencourt). Prior to sequencing, the samples were analyzed by a Bioanalyzer high-sensitivity DNA kit (Agilent) and quantified by quantitative PCR (qPCR) using the KAPA library quantification kit for Illumina. Pooled amplicon library samples were sequenced using Illumina NextSeq 500 with a single end 75-bp read configuration. Each sample was subjected to approximately 10 to 15 million reads. Raw FASTQ files were demultiplexed and trimmed to extract 20-bp sgRNA prior to mapping reads to reference Brunello sgRNA sequences. MAGeCK (18) software was used for data analysis; median normalization was used to adjust for the effect of library sizes and read count distribution. Positively selected sgRNAs and genes were identified with default parameters.

### Viral infection assays.

The cells were seeded at 80,000 to 100,000 cells/well to 24-well plates the day before infection. Virus inoculum (MOI 0.01 to 1) was added to each well in 200 μL of DMEM supplemented with 1% FBS for 0.5 to 3 h and then brought up to 500 μL volume with complete DMEM without or with inhibitor supplemented for the remaining incubation time. At the end of approximately one replication cycle as determined in pilot assays, the medium was aspirated, and the cells were lifted with Accumax (Sigma), mixed with paraformaldehyde (PFA) in PBS to a final concentration of 1% PFA, and fixed for 10 min at room temperature. Fixed GFP-virus-infected cells were pelleted and resuspended in PBS supplemented with 3% FBS prior to analysis on a S1000 (Stratedigm) flow cytometer, and the data were analyzed by FlowJo software. Nonreporter virus-infected cells were subjected to fixation and permeabilization (BD Cytofix/Cytoperm no. 554714), followed by antibody staining: DENV2 and ZIKV, anti-E protein D1-4G2-4-15; CoV OC43, anti-NP Millipore No. MAB9013; CoV SARS-CoV-2, anti-NP Sino Biological No. 40143-MM05; IAV, anti-NP HT103 Kerafast No. EMS010; Reovirus, anti-T3D G5 was deposited to the DSHB by T.S. Dermody (DSHB Hybridoma Product G5). Antibody-stained cells were resuspended in PBS supplemented with 3% FBS and analyzed by flow cytometry. For flavivirus viral production experiments, infections were performed as described above, except the viral inoculum was removed after 1 h, the cells were washed four times, and the supernatants were collected at the indicated time points and used for plaque assay or reinfection assay to quantify virus production. HCV-Gluc virus infections were performed with 40,000 cells/well in 48-well plates, the HCV-Gluc virus inoculum was removed, the cells were washed four times, and the supernatants were collected at the indicated time points and used for *Renilla* luciferase assays to quantify viral replication. For SCRPSY-TagRFP lentiviral transduction assays, 50,000 cells were seeded into 48-well plates, and lentivirus particles were added to cells in a dose-dependent manner. The cells were collected 24 h later, fixed in 1% PFA, and TagRFP expression was quantified by flow cytometry.

For Ad5-GFP dose-response experiments, the cells were seeded at 80,000 to 100,000 cells/well to 24-well plates the day before infection. Increasing doses of Ad5-GFP (up to 75 MOI) was added to each well in 200 μL of DMEM supplemented with 1% FBS for 1 h and then brought up to 1 mL volume with complete media. At the end of infection, the medium was aspirated, and the cells were lifted with Accumax (Sigma), mixed with PFA in PBS to a final concentration of 1% PFA, and fixed for 10 min at room temperature. Fixed GFP-virus-infected cells were pelleted and resuspended in PBS supplemented with 3% FBS prior to analysis on a S1000 (Stratedigm) flow cytometer, and the data were analyzed by FlowJo software.

For VEEV-GFP dose-response experiments, the cells were seeded at 80,000 to 100,000 cells/well to 24-well plates the day before infection. Increasing doses of VEEV-GFP (up to ~50 MOI) was added to each well in 200 μL of DMEM supplemented with 1% FBS for 40 min and then brought up to 500 μL volume with complete DMEM. At the end of infection, the medium was aspirated, and the cells were lifted with Accumax (Sigma), mixed with PFA in PBS to a final concentration of 1% PFA, and fixed for 10 min at room temperature. Fixed GFP-virus-infected cells were pelleted and resuspended in PBS supplemented with 3% FBS prior to analysis on a S1000 (Stratedigm) flow cytometer, and the data were analyzed by FlowJo software.

### Transfection studies (replicon, plasmid DNA).

HCV replicons expressing Gaussia luciferase (Bi-Gluc-JFH-SG) were described previously (50). Replicon RNA was transcribed from linearized template plasmids with T7 RiboMAX Express (Promega) and cleaned by MEGAclear (Ambion/ThermoFisher) according to the manufacturer’s instructions. Control and KO cells were seeded at 40,000 per 48-well and transfected with 200 μg RNA in 25 μL Opti-MEM using Trans-IT mRNA transfection kit (Mirus) according to the manufacturer’s instructions. At each time point, supernatants containing secreted Gluc were collected, and fresh medium was replaced until the end of the time course. Gluc *Renilla* luciferase units (RLUs) were measured in 10 μL of each supernatant mixed with 10 μL 2× *Renilla* lysis buffer (RLB) with a *Renilla* luciferase assay kit (Promega) in white 96-well plates on a Berthold plate reader. For plasmid transfection assays, 200 ng of plasmid pTagRFP-C (Evrogen), which expresses TagRFP from a CMV promoter, or 200 ng pSCRPSY-TagRFP lentiviral backbone plasmid were transfected into 50,000 cells in a 48-well plate using XtremeGene 9 according to the manufacturer’s instructions. The cells were collected 24 h later and fixed in 1% PFA, and TagRFP expression was quantified by flow cytometry.

### Viral titer by plaque assay.

Infectious YFV-17D particles were quantified by infecting serial 1:10 dilutions of supernatants on BHK-21J cells as previously described (61). IAV infectious particles were measured similarly by infecting serial 1:10 dilutions of supernatants on MDCK cells as previously described (54).

### Viral titer by supernatant reinfection.

Infectious DENV2 or ZIKV PRVABC159 particles were quantified by reinfecting 1:5-fold dilutions of supernatants on Huh7.5 cells seeded at 80,000 cells/well in 24-well plates. Supernatant-infected Huh7.5 cells were lifted with Accumax and fixed in 1% PFA 24 h postinfection. Infected cells were stained with anti-E protein D1-4G2-4-15 antibody prior to flow cytometry. The percentage of infected Huh7.5 cells per condition was used to calculate the proportion of infectious particles per volume of supernatant as previously described (62).

### Guide-resistant and site-directed mutant complementation constructs.

SBDS in pShuttle was purchased from GeneCopoeia (catalog No. GC-T0315). SPATA5 cDNA was generated from U-2 OS RNA using SuperScript IV first-strand synthesis system (Invitrogen), and SPATA5 was amplified from cDNA using *Ex Taq* (TaKaRa) with primers designed in-house (Table S3) that contain BP overhangs and recombined into pENTR using BP Clonase (ThermoFisher). Guide-resistant pENTR or pShuttle constructs were generated by modification of PAM sites by introducing silent mutations using site-directed mutagenesis (Table S3). SPATA5 pENTR construct was modified for both guide 1 and guide 2 PAM sites in each “dual” CRISPR KO treatment, whereas SBDS pENTR plasmid was modified only for one PAM site each because one sgRNA PAM site was outside the protein coding region. Silent site-directed PAM mutations were confirmed by sequencing, and guide-resistant constructs were moved to the blasticidin-selectable pSCRBBL lentivirus expression vector using Gateway LR clonase II (ThermoFisher). Guide-resistant pSCRBBL plasmids were used as templates for site-directed mutagenesis using oligonucleotides to introduce catalytic mutations (Table S3), and the resulting candidate plasmids were sequenced to confirm nucleotide changes. Blasticidin-selectable WT and mutant pSCRBBL vectors were used to generate infectious lentivirus and were transduced to KO cells generated by dual selection of guide 1 and guide 2 sgRNAs in LentiCRISPRv2-puromycin vectors to test complementation. For complementation experiments, Huh7.5 cells were transduced by plating cells in the presence of lentivirus expressing two different gene-targeting sgRNAs for 48 h, followed by selection. Seven days post-transduction, the cells were transduced again with SCRBBL lentivirus expressing guide-resistant complementation constructs for 48 h, followed by selection. Five days after complementation, 5,000 cells/well were plated in 96-well plates in a final volume of 100 μL media. The cells were harvested at 24, 48, and 96 h for WST-1 Assay (TaKaRa Bio) and Cell Titer Glo (Promega) following the manufacturer’s instructions. Alternatively, 70,000 cells were plated in 24-well plates, and the cells were infected by VEEV-GFP, followed by flow cytometry to assess viral infectivity.

### Western blots.

The cells were lysed using M-PER (ThermoScientific) supplemented with 1× protease inhibitor tablet (Roche) according to the manufacturer’s instructions. Lysates mixed with 2× Laemmli sample buffer (Bio-Rad) supplemented with β-mercaptoethanol were boiled for 5 min and separated on 12% SDS-PAGE gels prior to transfer to methanol-activated polyvinylidene difluoride (PVDF) membranes using the Trans-Blot Turbo system (Bio-Rad). The membranes were blocked in Tris-buffered saline with Tween 20 (TBS-T) and 5% dry milk and then probed with dilutions of primary and secondary horseradish peroxidase (HRP) antibodies (anti-SBDS Novus No. NBP2-22594, anti-SPATA5 Novus No. NBP2-38302, anti-actin HRP Sigma No. A3854, anti-mouse HRP, anti-rabbit HRP) in TBS-T and developed to X-ray film using Clarity Western ECL reagent (Bio-Rad). Blots reprobed with additional antibodies were first stripped with Re-blot Plus (Millipore No. 2504) solution and incubated with TBS-T plus milk blocking prior to follow-up primary and secondary antibody treatments.

### 5-EU labeling of rRNA.

Cells were seeded at 100,000/well in 24-well plates the day before, then incubated with 500 μM 5-EU (Click Chemistry Tools No. 1261) diluted in complete media for 2 h, rinsed, lifted with Accumax, and fixed in 1% PFA. Following fixation, the cells were permeabilized by PBS supplemented with 3% bovine serum albumin (BSA) and 0.1% saponin for 15 min. 5-EU was labeled in permeabilized cells using the Click & Go kit (Click Chemistry Tools No. 1263) with 2 μM Alexa Fluor 488 azide (Click Chemistry tools No. 1275) according to the manufacturer’s instructions. Labeled cells were washed twice and resuspended with PBS containing 3% FBS prior to analysis by flow cytometry.

### Drug inhibitor treatments.

The cells were seeded at 16,000 cells/well to 96-well plates the day before inhibitor addition. The medium was aspirated, and inhibitors were added at the following concentrations in a final volume of 100 μL of medium: 50 μM sodium arsenite, 2 μM emetine, 2 μM cyclohexamide, and 10 μM anisomycin. The cells were harvested at 24 h for Cell Titer Glo assay following the manufacturer’s instructions.

### Polysome fractionation.

KO or control Huh7.5 cells were seeded at 6.5 × 10^6^/15-cm plate 24 h prior to the experiment. Uninfected control or KO cells were lifted with trypsin and lysed in polysome lysis buffer (20 mM Tris-HCl, pH 7.4, 5 mM MgCl_2_, 100 mM NaCl, 1× protease inhibitor, 0.15% NP-40, 100 μg/mL cycloheximide, 1:400 RNasin). For infected cell polysome analysis, 15-cm plates of WT Huh7.5 cells were infected with ~MOI 5 YFV-17D or YFV-17D-Venus for 24 h prior to harvest. The infected cells were harvested and treated similarly, except the concentration of NP-40 in polysome lysis buffer was increased to 0.5% to inactivate virus. Cell lysates were clarified by centrifugation at 12,000 × *g* for 10 min at 4°C and layered on top of discontinuous gradients containing layers of 60% to 0% sucrose in 14 × 95-mm ultracentrifugation tubes. Gradients were centrifuged at 150,000 × *g* for 3 h at 4°C in a SW40ti rotor. Separated gradients were analyzed by A_254_ with a Bio-Rad BioLogic LP, and 500 μL fractions were collected with a Bio-Rad BioFrac.

### rRNA Northern blotting.

RNA from 5 × 10^6^ cells was extracted using 1 mL TRIzol according to the manufacturer’s instructions. A total of 10 μg of purified RNA was analyzed for rRNA processing by Northern blotting using NorthernMax kit (Invitrogen) and the probes listed in Table S3.

### Immunofluorescence for RSL24D1.

Control or KO cells were seeded in 8-well chamber slides (Falcon) overnight prior to fixation with 4% paraformaldehyde. The fixed cells were permeabilized with PBS plus 0.1% Triton X-100 for 10 min and blocked for 1 h with PBS containing 5% BSA and 0.1% Tween 20. Chamber slides were incubated with rabbit anti-RSL24D1 (ProteinTech) diluted 1:100 in PBS with 1% BSA and 0.3% Triton X-100 overnight at 4°C. The next day chamber slides were washed with PBS, counterstained with donkey anti-rabbit Alexa Fluor 488 (Invitrogen) for 30 min, washed three times with PBS, and mounted with Vectashield containing 4′,6-diamidino-2-phenylindole (DAPI). The cells were imaged with a Zeiss LSM780 inverted confocal microscope and analyzed by FIJI. FIJI quantifications were done with a previously published FIJI macro to determine the cytoplasmic and nuclear protein distributions (63). Quantifications were done with three separate transduction replicates of cells except SBDS, which was done with two separate transductions. At least six image fields were quantified per cell line per replicate. On average, each image field contained 40 cells. The data are shown as one dot corresponding to the average cytoplasmic localization of one image field with bars showing the average of all image fields quantified.
