# Supplementary figures and images for: Genome-Scale CRISPR Screening Reveals Host Factors Required for Ribosome Formation and Viral Replication
Source: mBio. 2023 Feb 21;14(2):e00127-23. doi: 10.1128/mbio.00127-23 (PMC10128003; doi:10.1128/mbio.00127-23)

**A**

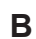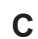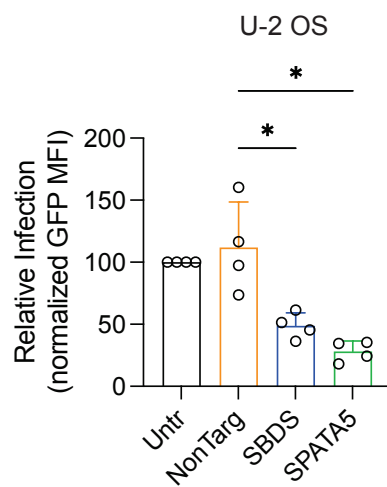

Supplement: FIG S1 [file mbio.00127-23-s0004.pdf]

Figure S2

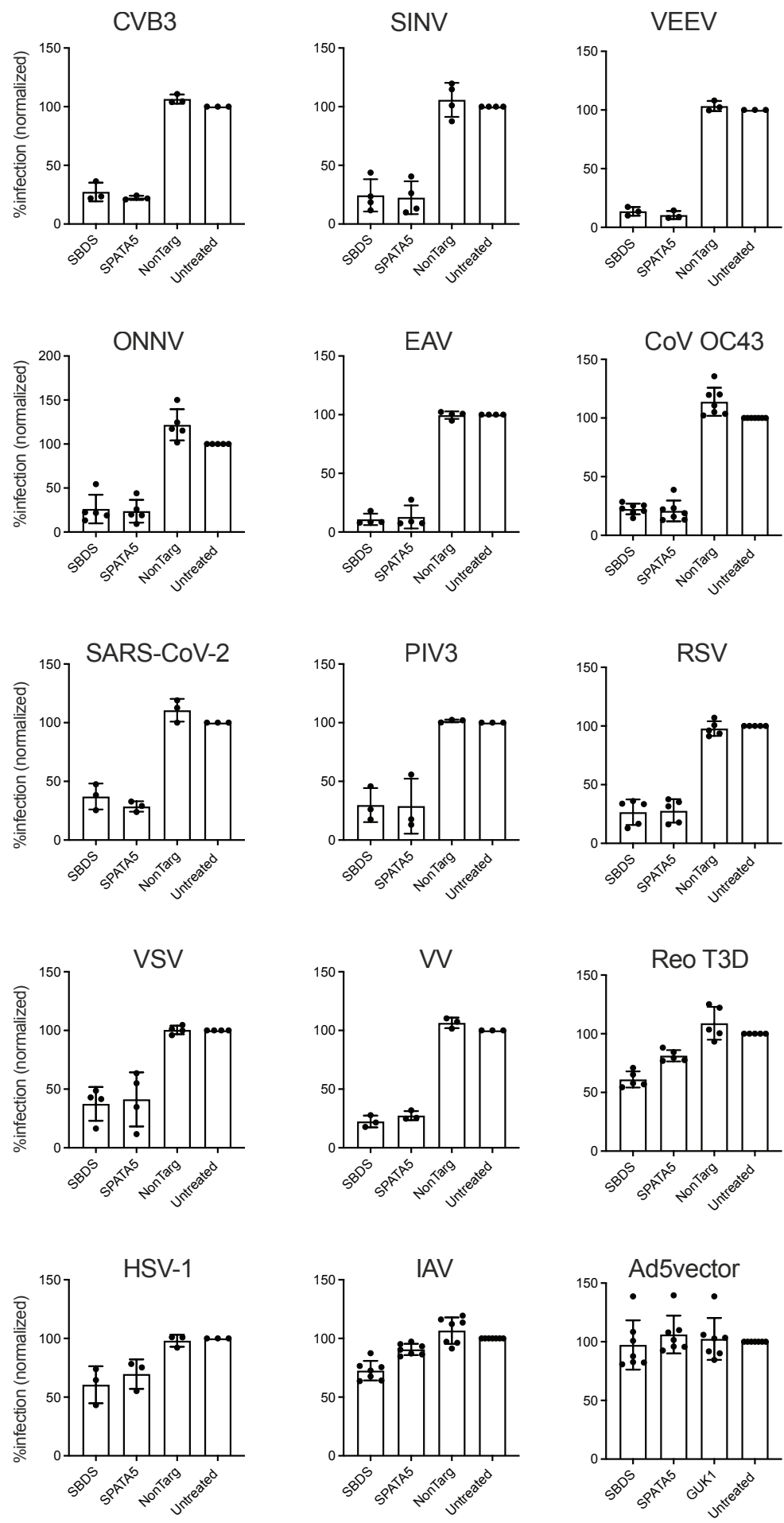

Supplement: FIG S2 [file mbio.00127-23-s0005.pdf]

Figure S3

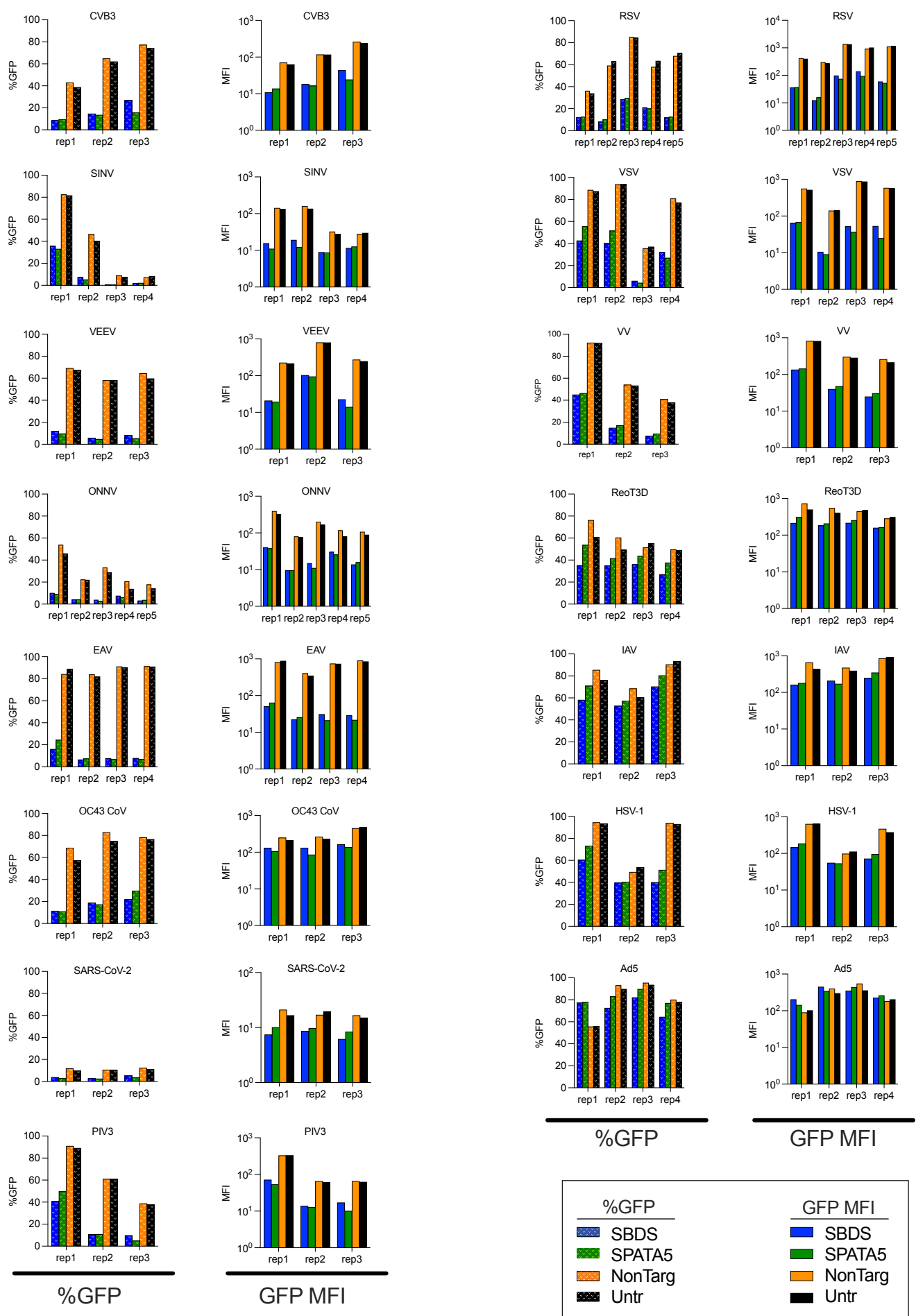

Supplement: FIG S3 [file mbio.00127-23-s0006.pdf]

Figure S4

A

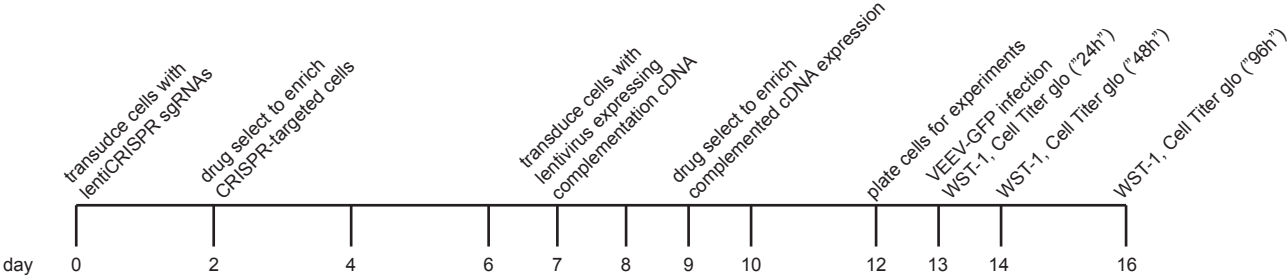

B

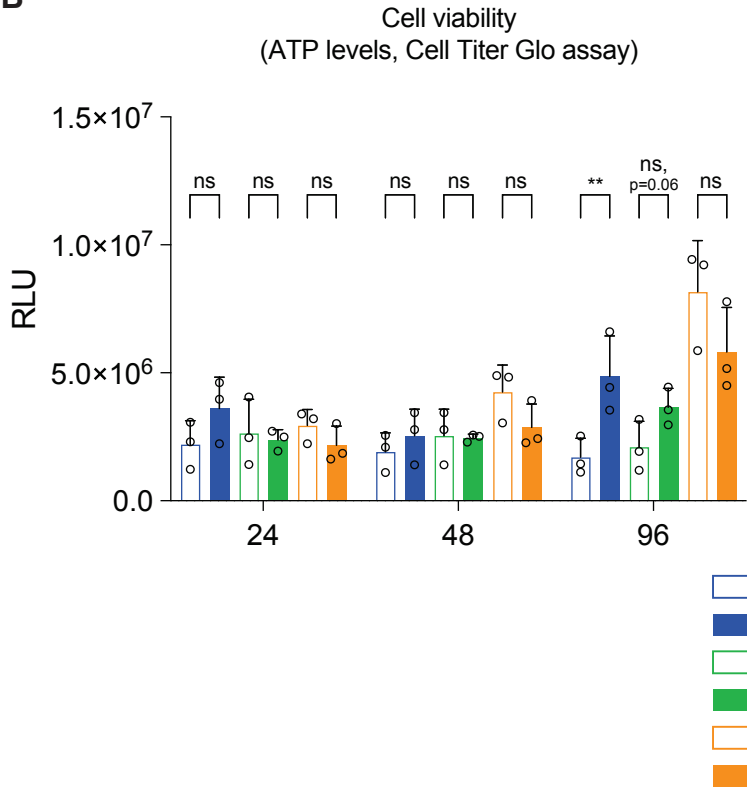

C

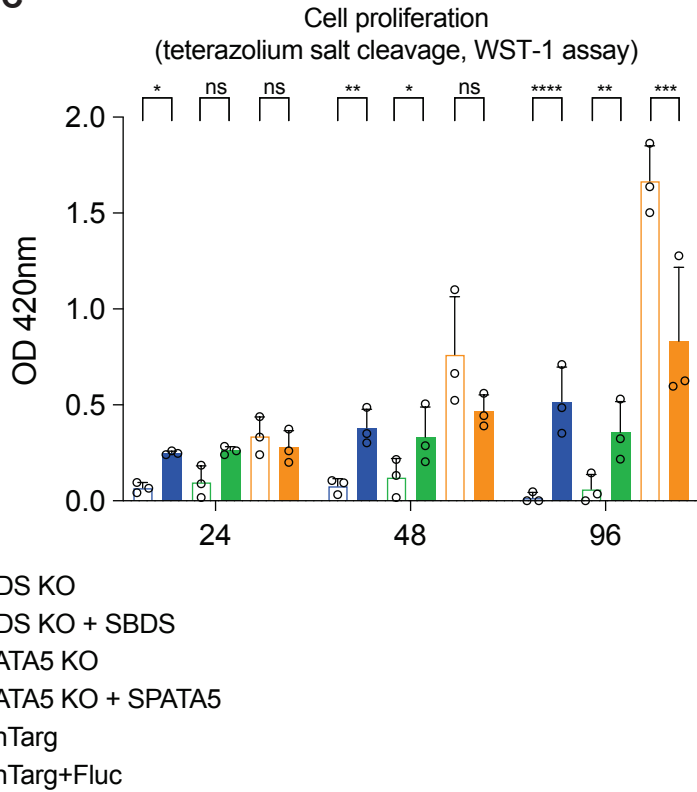

D

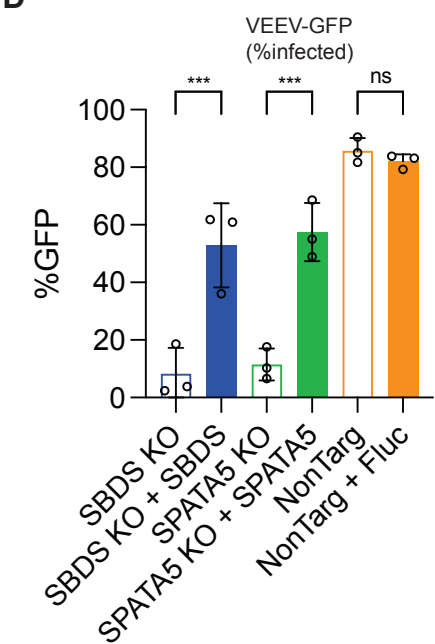

E

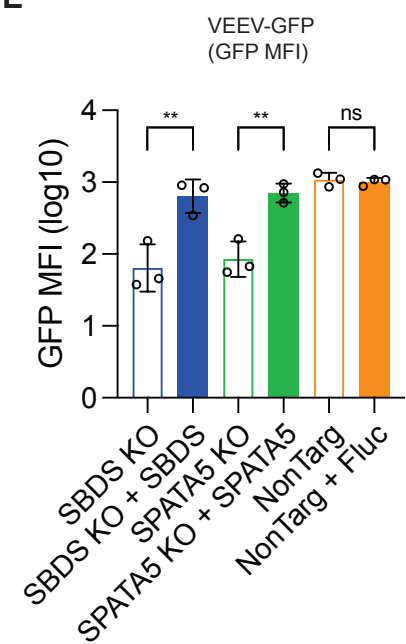

Supplement: FIG S4 [file mbio.00127-23-s0007.pdf]

Figure S5

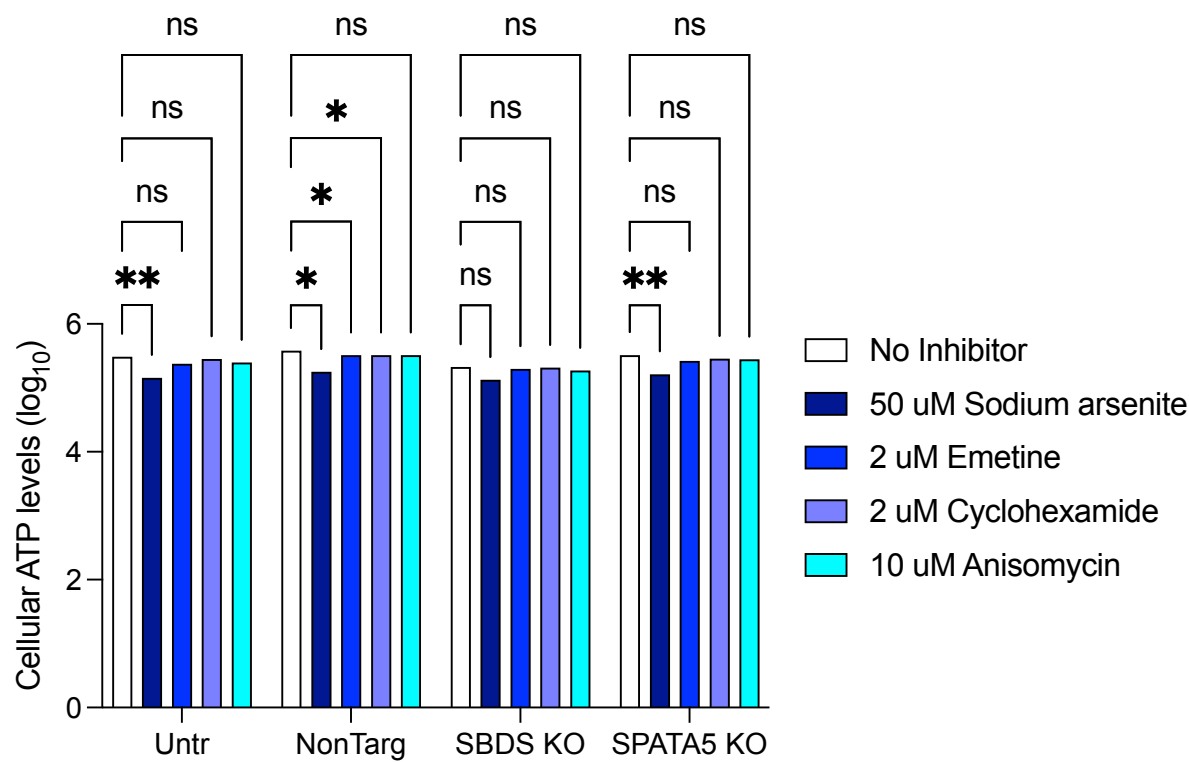

Supplement: FIG S5 [file mbio.00127-23-s0008.pdf]

Figure S6

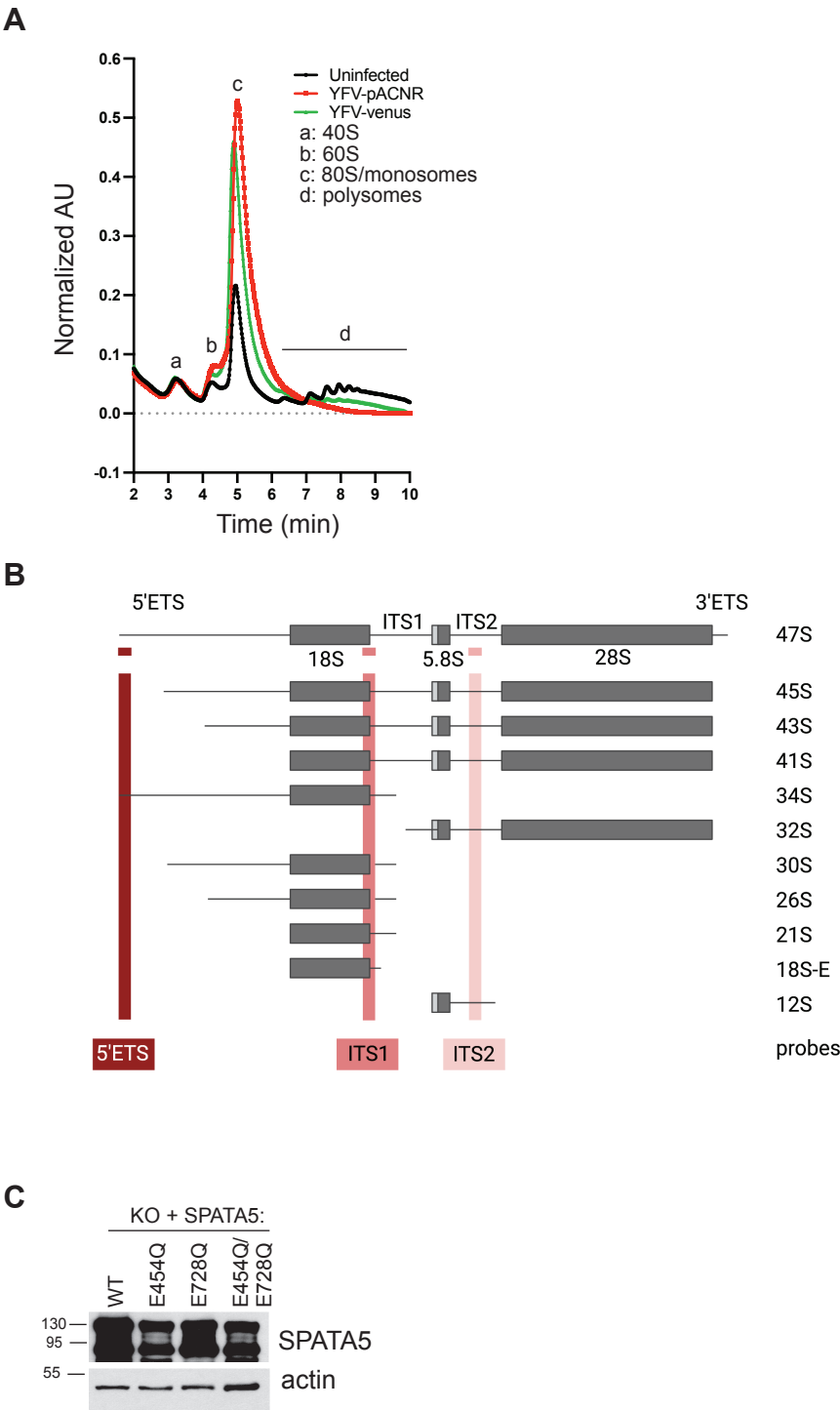

Supplement: FIG S6 [file mbio.00127-23-s0009.pdf]
