# Supplementary material for: Genome-Scale CRISPR Screening Reveals Host Factors Required for Ribosome Formation and Viral Replication
Source: mBio. 2023 Feb 21;14(2):e00127-23. doi: 10.1128/mbio.00127-23 (PMC10128003; doi:10.1128/mbio.00127-23)
Supplement: TABLE S2 [file mbio.00127-23-s0002.pdf]

Table S2. Viruses tested for replication in Huh7.5 host factor knockout cells

**Virus name (abbreviation)**	**Family**	**Genome**
Yellow fever virus (YFV)	Flaviviridae	+ssRNA
Zika virus (ZIKV)	Flaviviridae	+ssRNA
Dengue virus (DENV)	Flaviviridae	+ssRNA
West Nile virus (WNV)	Flaviviridae	+ssRNA
Hepatitis C virus (HCV)	Flaviviridae	+ssRNA
Coxsakie virus B (CVB)	Picornaviridae	+ssRNA
Equine arteritis virus (EAV)	Artirivirus	+ssRNA
Sindbis virus (SINV)	Togaviridae	+ssRNA
Venezuelan equine encephalitis virus (VEEV)	Togaviridae	+ssRNA
O'nyong'nyong virus (ONNV)	Togaviridae	+ssRNA
Coronavirus (CoV OC43)	Coronaviridae	+ssRNA
Coronavirus (SARS-CoV-2)	Coronaviridae	+ssRNA
Parainfluenza virus 3 (PIV3)	Paramyxoviridae	-ssRNA
Respiratory syncytial virus (RSV)	Paramyxoviridae	-ssRNA
Vesicular stomatitis virus (VSV)	Rhabdoviridae	-ssRNA
Reovirus	Reoviridae	dsRNA
Influenza A (FluA)	Orthomyxoviridae	-ssRNA
Adenovirus 5 (Ad5)	Adenoviridae	dsDNA
Herpes simplex virus 1 (HSV-1)	Herpesviridae	dsDNA
Vaccinia virus (VV)	Poxviridae	dsDNA
